# In vitro interactions between *Bradyrhizobium* spp. and *Tuber magnatum* mycelium

**DOI:** 10.1111/1758-2229.13271

**Published:** 2024-05-01

**Authors:** Simone Graziosi, Federico Puliga, Mirco Iotti, Antonella Amicucci, Alessandra Zambonelli

**Affiliations:** ^1^ Department of Agricultural and Food Sciences University of Bologna Bologna Italy; ^2^ Department of Life, Health and Environmental Science University of L'Aquila L'Aquila Italy; ^3^ Department of Biomolecular Sciences University of Urbino Urbino Italy

## Abstract

*Tuber magnatum* is the most expensive truffle, but its large‐scale cultivation is still a challenge compared to other valuable *Tuber* species. *T. magnatum* mycelium has never been grown profitably until now, which has led to difficulties to studying it in vitro. This study describes beneficial interactions between *T. magnatum* mycelium and never before described bradyrhizobia, which allows the in vitro growth of *T. magnatum* mycelium. Three *T. magnatum* strains were co‐isolated on modified Woody Plant Medium (mWPM) with aerobic bacteria and characterised through microscopic observations. The difficulties of growing alone both partners, bacteria and *T. magnatum* mycelium, on mWPM demonstrated the reciprocal dependency. Three bacterial isolates for each *T. magnatum* strain were obtained and molecularly characterised by sequencing the 16S rRNA, *glnII*, *recA* and *nifH* genes. Phylogenetic analyses showed that all nine bacterial strains were distributed among five subclades included in a new monophyletic lineage belonging to the *Bradyrhizobium* genus within the *Bradyrhizobium jicamae* supergroup. The *nifH* genes were detected in all bacterial isolates, suggesting nitrogen‐fixing capacities. This is the first report of consistent *T. magnatum* mycelium growth in vitro conditions. It has important implications for the development of new technologies in white truffle cultivation and for further studies on *T. magnatum* biology and genetics.

## Graphical abstract

*Tuber magnatum* mycelium, together with its associated bacteria (*Bradirhizobium* spp.), were isolated for the first time. Cultural and phylogenetic analyses revealed the specificity of this interaction.

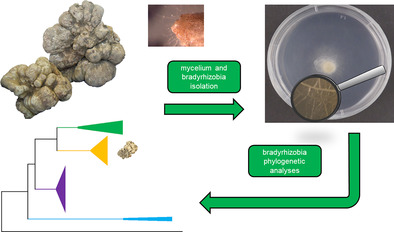

## INTRODUCTION

True truffles are hypogeous ascomycetes within the genus *Tuber*. This genus includes more than 180 ectomycorrhizal species (Bonito et al., 2013), and some of them have the highest economic value among edible mushrooms (Luxury Columnist, 2022) due to their excellent organoleptic properties (Mello et al., 2006). *Tuber magnatum* Picco, *Tuber melanosporum* Vittad., *Tuber aestivum* Vittad., and *Tuber borchii* Vittad. are the most economically important species, but only the last three have been extensively cultivated until now. Their cultivation is achieved by planting truffle seedlings in appropriate soils and climates. The most common inoculum type used by nurseries to produce truffle seedlings is made by crushing fresh, frozen or dried fruiting bodies to obtain a spore slurry that is used to inoculate the root system (Iotti, Piattoni, & Zambonelli, 2012). Several authors demonstrated that it is also possible to produce *Tuber* mycorrhizas with mycelial cultures (Chevalier & Frochot, 1997; Sisti et al., 1997) and that the truffle plants obtained by mycelial inoculum can fructify like those obtained by spore inoculum (Iotti et al., 2016). However, not all truffle mycelia can be cultivated in vitro conditions and, when possible, they grow slower than the mycelia of other ectomycorrhizal species. *T. borchii* (Barbieri et al., 2005), *Tuber rufum* Picco, and *Tuber macrosporum* Vittad. (Iotti et al., 2002) are some of the species that can be successfully isolated and grown on agar media, whereas *T. magnatum* mycelium is hard to isolate and its development has been limited to a few hundred micrometres (Iotti, Piattoni, & Zambonelli, 2012). Fontana (1968) first reported the isolation of *T. magnatum* mycelium from a fragment of gleba transferred on agar plates. Later, Mischiati and Fontana (1993) affirmed that they had isolated *T. magnatum* mycelium from mycorrhizas, but several years later, Mello et al. (2001) genetically verified that this mycelium belonged to the whitish truffle *Tuber maculatum* Vittad.

During their development in soil, the mycelium of ectomycorrhizal fungi interacts with many microorganisms, some of which, as the mycorrhiza helper bacteria, may affect the fungal metabolism and growth (Frey‐Klett et al., 2007). Bacteria also play an essential role in the life cycle of truffles, and in particular, the microbiome associated with *T. magnatum* ascomata seems to have a crucial role in aroma biosynthesis (Vahdatzadeh et al., 2015), fruiting body formation and nutrition (Monaco et al., 2022).

Many taxa of bacteria live in the ascoma of *T. magnatum* (Barbieri et al., 2007, 2010; Citterio et al., 1995; Monaco et al., 2021; Niimi et al., 2021a) and other *Tuber* spp. The majority of them belong to *Proteobacteria*, in particular *Gammaproteobacteria* and *Alphaproteobacteria*. The last includes *Bradyrhizobium*, which is the most abundant bacterial genus found in truffle ascomata (Antony‐Babu et al., 2014; Benucci & Bonito, 2016; Niimi et al., 2021b; Sillo et al., 2022). Barbieri et al. (2010) hypothesised the involvement of *Bradyrhizobium* in the nitrogen nutrition of *T. magnatum*. They detected the nitrogenase gene *nifH* of *Bradyrhizobium* spp. inside the *T. magnatum* ascoma and found that the level of nitrogen fixation was comparable to that of early nodules of legumes associated with specific nitrogen‐fixing bacteria (Barbieri et al., 2010, 2012).

Until now, the role of the *Bradyrhizobium* spp. or other Proteobacteria living inside *T. magnatum* ascomata on its mycelium development in vitro conditions has never been investigated. However, a few years ago, Le Roux et al. (2016) identified Alphaproteobacteria belonging to *Rhodopseudomonas* growing associated with the mycelia of *T. melanosporum* and *T. brumale*, which seemed to maintain the vitality of these truffle mycelia after repeated subculturing. Since *Tuber* mycelia grow slowly in vitro conditions and the risk of losing them after the first subculture is very high (Giomaro et al., 2005), the improvement of their growth performances would be fundamental for both scientific studies and truffle cultivation applications.

In this work, we isolated and maintained in vitro the mycelium of *T. magnatum* for the first time thanks to the presence of *Bradyrhizobium* spp. living inside the ascoma. These bacteria were characterised by phylogenetic analyses of four genes, and their specificity for *T. magnatum* was assessed by co‐culture tests with other *Tuber* species.

## EXPERIMENTAL PROCEDURES

### Mycelium isolation

During 2021 and 2022, many attempts to isolate *T. magnatum* strains from fresh ascomata collected in Italy were carried out. Fragments of gleba, 1–2 mm in size, were aseptically excised from the inner part of the ascoma and cultured in Petri dishes on modified Woody Plant Medium (mWPM) (Iotti et al., 2005) at 22.5°C in the dark. Each ascoma was then dried and deposited in the herbarium of the ‘Centro di Micologia’ of Bologna (CMI‐UNIBO) (Table 1). All isolates were subcultured every 50–60 days on mWPM to stabilise the cultures.

**TABLE 1:** List of *Tuber magnatum* ascomata used for mycelium isolation.

Strain	Species	Putative host	Provenience^a^	Date
TMG5072^b^	*T. magnatum*	na	Molinella (BO), Emilia Romagna, Italy	28 September 2021
TMG5299	*T. magnatum*	na	na	14 September 2022
TMG5300^b^	*T. magnatum*	na	na	14 September 2022
TMG5301	*T. magnatum*	na	na	14 September 2022
TMG5302	*T. magnatum*	na	na	14 September 2022
TMG5312	*T. magnatum*	*Populus alba* L.	Montefalcone nel Sannio (CB), Molise, Italy	15 November 2022
TMG5316	*T. magnatum*	na	na	09 November 2022
TMG5317	*T. magnatum*	na	na	09 November 2022
TMG5318	*T. magnatum*	na	na	09 November 2022
TMG5319^b^	*T. magnatum*	*Quercus cerris* L.	Città della Pieve (PG), Umbria, Italy	15 November 2022

Abbreviations: BO, Bologna; CB, Campobasso; na, not available; PG, Perugia.

^a^ na = provided by Truffleland s.r.l, Sant'Anatolia di Narco, Perugia, Italy.

^b^ Strain with an active growing mycelium.

The identity of each *T. magnatum* isolate was molecularly confirmed by polymerase chain reaction (PCR) with the species‐specific primers *TmgI* and *TmgII* (Amicucci et al., 1998). All the PCR reactions were carried out by mixing 25 μL of 2× Phanta® Max Master Mix (Vazyme Biotech Co) with 2 μL of each primer (10 μM), 1 μL of 2.5% dimethylsulphoxide and nuclease‐free sterile water to a reaction volume of 50 μL. Some hyphae were put directly into the reaction volume in aseptic conditions. The reaction mixtures underwent an initial denaturation step of 94°C for 5 min, followed by 25 cycles of 20 s at 94°C, 15 s at 62°C, 1 min at 72°C and a final extension at 72°C for 7 min. PCR products were run on 1% agarose gel and visualised by staining with ethidium bromide.

### Microscopic observations

The morphological characteristics of the hyphae of *T. magnatum* isolates were observed and measured under a Nikon Eclipse TE2000 U Inverted Microscope (Nikon Corporation, Tokyo, Japan) and images captured with a Nikon DS‐Fi3 (Nikon Corporation, Tokyo, Japan). The measures were collected with the NIS Elements BR software (ver. 4.6, Nikon Corporation, Tokyo, Japan).

*T. magnatum* hyphae from the inner part of the colony of each strain were first observed without any treatments and then observed again after washing in sterile water added with 0.5% Tween 20, followed by vortexing for 1 min to remove loosely attached bacteria. Blue lactophenol was used to stain the wall polysaccharides of hyphae and Gram‐negative bacteria cells (Ericksen, 2015).

The hyphal features selected to describe *T. magnatum* strains were hyphal diameter, septal distance, Hyphal Growth Unit (HGU) (Trinci, 1974) and Vesicle Production Ratio (VPR). Vesicles are common morphological features of *Tuber* spp. mycelium and are represented by hyphal swellings (Iotti et al., 2002). VPR is represented by the formula:

$$\mathrm{VPR}=\frac{\;\text{vesiscle number}}{\text{septal distance}}.$$

### Antibiotic treatment

At first, preliminary analysis was carried out on *T. borchii*—CMI‐UNIBO, strain n. TBO5005 (Puliga et al., 2021)—to evaluate the effect of antibiotic addition on *Tuber* spp. mycelial growth. To this purpose, 200 μg/mL of streptomycin, ampicillin and chloramphenicol (Kuykendall et al., 1988) were added to mWPM plates. Five mWPM plates (9 cm in diameter) added with antibiotics were inoculated with 0.5 cm mycelium plugs from 50‐day‐old *T. borchii* cultures, and the other five mWPM plates without antibiotic addition were used as controls. The colony diameter of each plate was measured every 7 days until the stationary phase (10 weeks), along two preset diametrical lines. After this preliminary analysis, the same procedure was applied to evaluate the mycelial growth of *T. magnatum* in the absence of bacteria. *T. magnatum* mycelial plugs were taken from 60‐day‐old cultures of each fungal isolate containing wild bacteria strains.

The area (cm^2^) covered weekly by the mycelium was calculated according to Puliga et al. (2022), assuming an elliptical shape covered by the mycelium as reported by Tryfinopoulou et al. (2020) with the following formula:

$$\mathrm{FCA}=R_{1}\times R_{2}\times \pi ,$$

where FCA is the fungal colony area (cm^2^) and *R*_1_ and *R*_2_ are the two perpendicular radii, respectively.

The area growth rate of the mycelium (AGR) was calculated with the formula of Sinclair and Cantero (1989):

$$\mathrm{AGR}=\frac{{\mathrm{FCA}}_{f}-{\mathrm{FCA}}_{i}}{T_{f}-T_{i}}$$

where FCA_f_ and FCA_i_ are the FCAs at the end and beginning of the exponential growth phase, respectively; *T*_f_ and *T*_i_ are the times (weeks) at the end and beginning of the exponential growth phase, respectively.

### Tuber *mycelia/bacteria co‐culture test*

The growth of the three *T. magnatum* strains (TMG5072, TMG5300 and TMG5319) was determined by measuring the FCA with the same method described above (Puliga et al., 2022). The bacterial population of the strain with the faster growth was selected for the next test.

The ability of the bacterial community isolated from *T. magnatum* ascomata to promote the growth of *Tuber borchii* (TBO5005), and *T. melanosporum*—CMI‐UNIBO, strain n. TME2 (Iotti, Rubini, et al., 2012)—was evaluated by co‐culture tests in mWPM plates (Iotti et al., 2005). For each truffle species, five plates (6 cm in diameter) were inoculated with 0.5 cm mycelium plugs and 10 μL of bacterial suspension (~1 × 10^8^ CFU/mL of the TMG5072 bacterial community) in yeast mannitol medium (YM) (Keele Jr et al., 1969). An additional five Petri dishes for each truffle species were inoculated only with the *Tuber* spp. mycelium as a control, added with 10 μL of liquid YM. Finally, other five Petri dishes were inoculated with 0.5 cm mycelium plugs of TMG5072 together with its native bacteria. The mycelium diameter of each strain was measured every 7 days until the stationary phase (10 weeks from inoculation) along two preset diametrical lines. FCA and AGR were calculated as previously reported.

### Statistical analyses

The morphological data, FCA and AGR were analysed using R Studio 2023.09.1+494. The significant difference between treatments was tested by one‐way analysis of variance and the means were compared by Tukey's *t*‐test (*p* ≤ 0.05).

### Bradyrhizobium *isolation*

The bacterial populations growing together with each isolated *T. magnatum* strain were transplanted into mWPM (Iotti et al., 2005) and yeast mannitol agar (YMA) and kept at both 22.5°C and 28°C in the dark.

*Bradyrhizobium* strain isolation and purification were made by streak‐planting (Sanders, 2012) on YMA at 28°C in the dark, which are the best conditions for *Bradyrhizobium* growth (Hafiz et al., 2021; Vincent, 1970). The isolation was carried out starting from the bacterial population of each fungal strain.

After bacterial growth, the identity of at least 10 colonies from each *T. magnatum* strain was verified by a direct PCR approach using the *Bradyrhizobium* spp.‐specific primers *BRdnaKf*–*BRdnaKr* (Menna et al., 2009). PCR reactions were performed with the thermal parameters specified in Table S1. Three bradyrhizobial colonies from each *T. magnatum* strain were randomly selected for phylogenetic analyses.

The isolated *Bradyrhizobium* strains were then transferred into YM liquid medium and grown on an orbital shaker at 180 rpm, at 28°C in the dark for 10 days (Iturralde et al., 2020). After that, 500 μL of these cultures were added with 500 μL of glycerol and preserved at –80°C.

### Phylogenetic analyses

Phylogeny of bradyrhizobia strains was inferred by maximum likelihood (ML) and neighbour joining (NJ) in raxmlGUI 1.5b2 (Silvestro & Michalak, 2012) and MEGA11 software (Tamura et al., 2021) using the genes 16S rRNA, *glnII*, *recA* and *nifH*. The selected genes were amplified through direct PCR using the primer pairs and the conditions reported in Table S1. The *nifH* gene was amplified with the newly designed primers *NifseqF* (ATTCTGATCGTCGGTTGCG) and *NifseqR* (GGATCTTCTCGGCAAGGC) to avoid non‐specific amplicons. Amplified fragments were sequenced at Eurofins Genomics (Germany) in both directions. Sequences were edited and assembled by the Bioedit Sequence Alignment Editor (Hall et al., 2011) and then aligned with the MUSCLE algorithm implemented in MEGA11 software (Tamura et al., 2021). The sequences were deposited in GenBank, and their accession numbers were listed here: OR544965–OR544973 (16S rRNA), OR569722–OR569730 (*glnII*), OR569731–OR569739 (*recA*) and OR569740–OR569748 (*nifH*). For each accession number, the closest BLASTn result was reported in Table S2.

Single gene phylogenies were inferred for the 16S rRNA, *glnII*, *recA* and *nifH* gene sequences, while a concatenated dataset was generated with the sequences of *glnII* and *recA* genes (Chahboune et al., 2011; Delamuta et al., 2017). The sequences used to construct phylogenetic trees and the outgroup are listed in Table S3. ML analyses were performed with 1000 throughout bootstrap replicates (100 runs), applying the models of nucleotide substitution GTR + G + I either for the 16S rRNA and *nifH* genes or for *glnII* + *recA* concatenated dataset. Single gene phylogenies of *glnII* and *recA* were inferred with NJ analysis with 1000 throughout bootstrap replicates (100 runs) and applying the *p*‐distance model. ML and NJ trees were edited using MEGA11 (Tamura et al., 2021). Only bootstrap values greater than 75% were shown on branches. Genetic diversity (*p*‐distance) within and among the *Bradyrhizobium* supergroups of both *glnII* and *recA* genes was evaluated using MEGA11 (Tamura et al., 2021).

## RESULTS

### Mycelium isolation

The PCR with specific primers confirmed the identity of all *T. magnatum* isolates, which were characterised by active and consistent growth in subsequent subcultures (TMG5072, TMG5302 and TMG5319). The mycelia grew both on the surface and in the agar medium. After the isolation procedure, all strains showed a very long lag phase. The excised fragments of the gleba also took more than 1 month to generate the first hyphae. The same behaviour was also observed after the first subculturing. The growth rate seemed to increase in the following subcultures, although the inocula took 2–3 weeks to form an evident hyphal extension from the plug. On mWPM, *T. magnatum* strains generally take 10 weeks from inoculation to reach the stationary phase.

**FIGURE 1:**
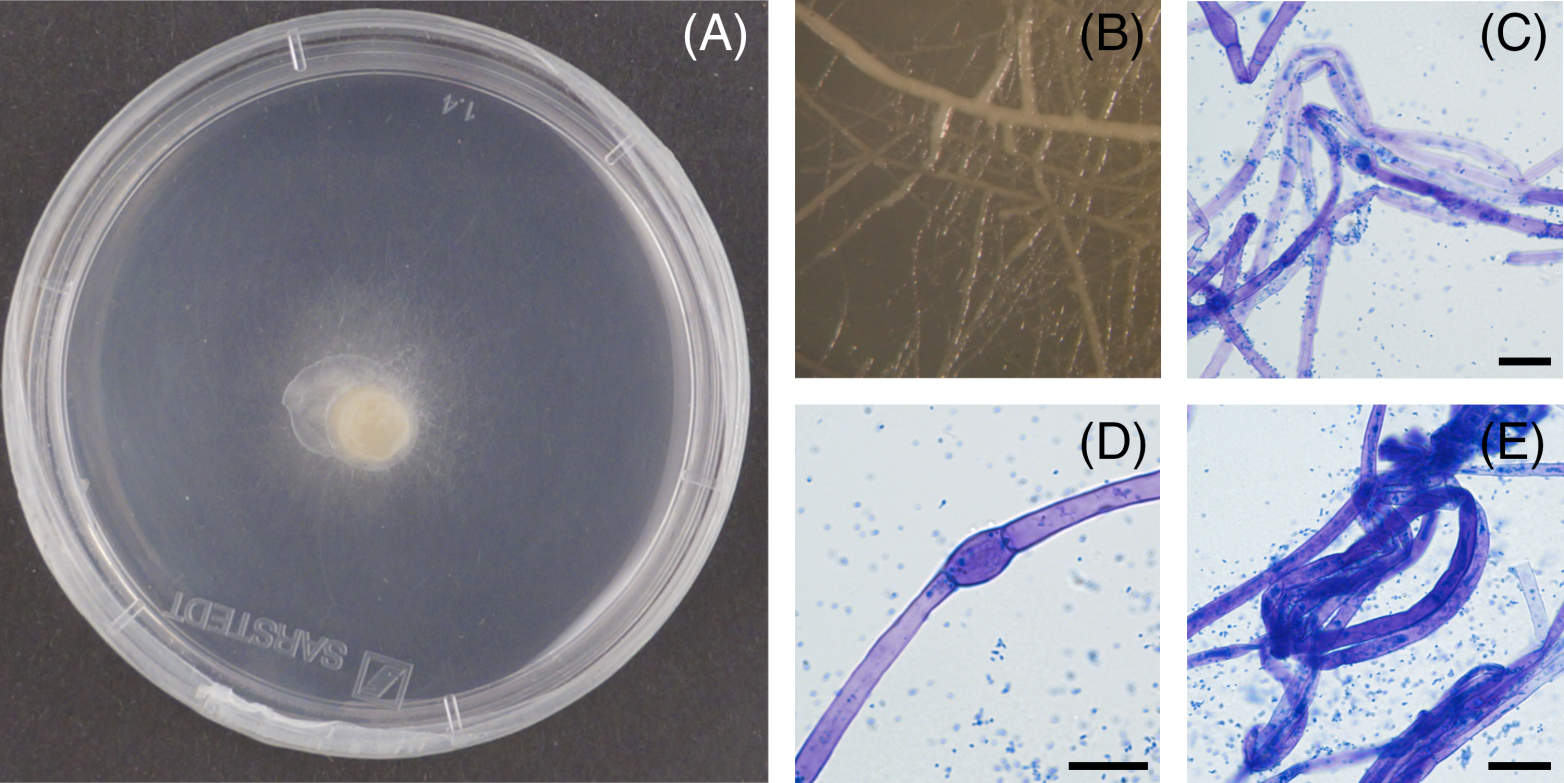
Example of *Tuber magnatum* mycelium culture on modified Woody Plant Medium (mWPM) at the first stage of growth (A). Bacterial biofilm around hyphae (B). Morphology of *T. magnatum* mycelium (C–E); bar = 15 μm. Bacterial distribution along the hypha (C), vesicles (D) hyphal coils and aggregates (E). Hyphae and bacteria were coloured with blue lactophenol staining.

The mycelial colony appeared whitish at the beginning (Figure 1) and gradually changed to ivory and pale yellow 8–10 weeks after inoculation.

Each mycelial isolate showed the co‐occurrence of a native bacterial population. During subcultures, the first days after inoculation, the bacteria grew around the inoculation point, forming a cream colony that remained circumscribed only in the inner area of mycelium growth. After the mycelium colonised almost all of the surface of the plate, the bacteria spread deep into the medium, which became a little opaque.

### Microscopic observations

For each strain, several vesicles, rare anastomoses and hyphal coils were observed (Graziosi et al., 2022) (Figure 1). The hyphal diameter averaged from 4.45 ± 0.16 μm for the strain TMG5072 to 5.38 ± 0.19 μm for the strain TMG5300 (Table 2). The statistical analysis of hyphal diameter showed no significant differences between the strains TMG5072 and TMG5319, whereas it was found between the strain TMG5300 and the other two strains.

**TABLE 2:** Hyphal morphological characteristics of the isolates.

Strain^1^	Hyphal diameter (μm)	Septal distance (μm)	Branching angle (°)	HGU (μm/no. of branches)	VPR (μm/no. of vesicles)
TMG5072	4.45 ± 0.16a (2.53–7.98)	56.86 ± 4.34a (5.56–149.85)	61.80 ± 7.31 (34–90)	144.81 ± 24.53 (50.17–220.01)	63.96 ± 9.76 (17.33–105.61)
TMG5300	5.38 ± 0.19b (3.31–8.70)	45.56 ± 6.44b (12.59–93.79)	67.70 ± 8.56 (34–112)	178.50 ± 37.76 (54.23–441.71)	66.77 ± 12.30 (32.24–113.11)
TMG5319	4.59 ± 0.13a (2.78–6.26)	55.49 ± 3.10c (16.42–126.04)	68.70 ± 7.69 (24–92)	145.62 ± 8.09 (104.5–175.94)	76.35 ± 7.78 (42.15–140.32)

*Note*: Data are the mean of 50 measures from three different Petri dishes. Within columns, different letters indicate difference between treatments according to *p* < 0.05 by Tukey's test.

Abbreviation: HGU, Hyphal Growth Unit.

^1^ Strain of *Tuber magnatum* used in this study.

The average septal distance ranged from 45.56 ± 6.44 for the strain TMG5300 to 56.86 ± 4.34 μm for the strain TMG5072, and statistical differences were observed among all the strains (Table 2).

The branching angle was very similar among the tested strains and no statistical differences were found. Strain TMG5072 showed the lowest average angle (61.80 ± 7.31°), whereas strain TMG5319 exhibited the greatest (68.70 ± 7.69°).

Regarding HGU, strain TMG5300 is characterised by the highest average value (178.50 ± 37.76 μm) in contrast with the TMG5072 and TMG5319 strains, which showed 144.81 ± 24.53 and 145.62 ± 8.09 μm, respectively. Thus, strain TMG5300 developed more linear hyphae and the lowest number of branches. Nevertheless, there were no statistical differences among strains.

The occurrence of vesicles was very similar among strains and no statistical differences were detected. Strain TMG5072 had the highest frequency of vesicles, with a VPR average value of 63.96 ± 9.76 μm followed by TMG5300 (66.77 ± 12.30 μm) and TMG5319 (76.35 ± 7.78 μm).

After staining, bacterial cells adhering to hyphae were evident, although it did not occur extensively along their entire length. Other bacterial cells remained spread in the cultural medium. After the washing treatment, all *T. magnatum* strains exhibited only some bacterial clusters that remained attached to the hyphae.

### Antibiotic treatment and co‐culture test

The growth of TBO5005 mycelium was not significantly affected by antibiotic addition (Figure S1), whereas TMG5072 mycelium and the associated bacteria were completely inhibited.

Among *T. magnatum* isolates, the strain TMG5072 showed the fastest growth and maintained its vitality with subculturing (Figure S2). On the contrary, mycelia of strains TMG5300 and TMG5319 grew slower and weaker just after the first subculture.

**FIGURE 2:**
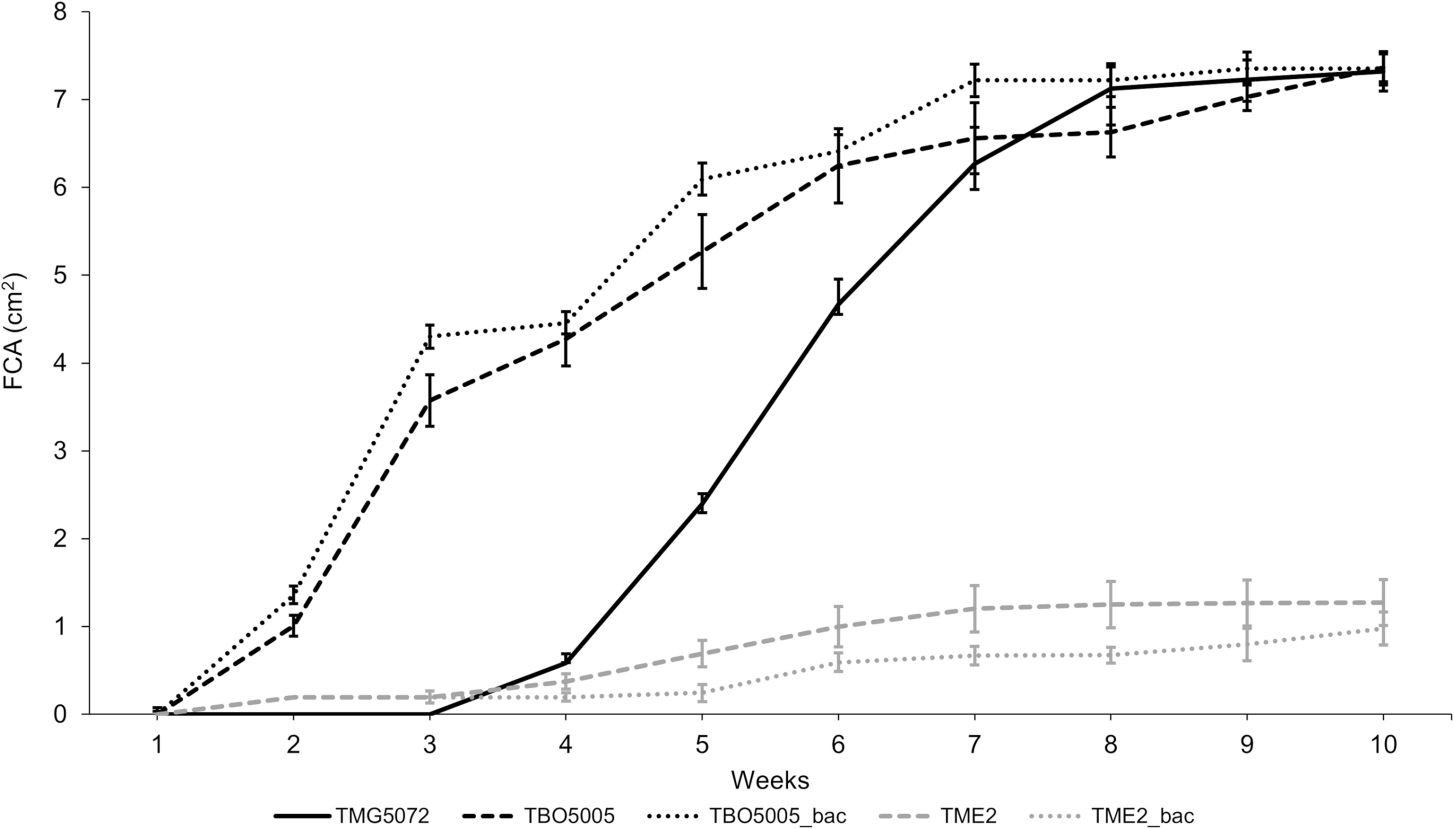
Growth trend of area covered weekly by mycelia of *Tuber magnatum* strain 5072 (TMG5072, black solid line), *Tuber borchii* control (TBO5005, black dashed line), *T. borchii* with bacterial addition (TBO5005_bac, black dotted line), *Tuber melanosporum* control (TME2, grey dashed line), *T. melanosporum* with bacterial addition (TME2_bac, grey dotted line). FCA, fungal colony area.

The FCA covered weekly by the tested truffle species on mWPM in the co‐culture test is reported in Figure 2. The lag‐phases differed between *Tuber* species (from a few days to 3 weeks). *T. borchii* was the first species to grow new hyphae, just 1 week after inoculation. *T. magnatum* and *T. melanosporum* were characterised by the longest lag‐phases and the exponential growth phase started 4 weeks after inoculation. Nevertheless, TMG5072 showed significantly faster growth and within 5 weeks after inoculation, its mycelial area exceeded that of TME2 (1.27 ± 0.26 cm^2^). At the stationary phase (8 weeks after inoculation), the area covered by TMG5072 mycelium (7.32 ± 0.23 cm^2^) reached approximately the TBO5005 one (7.36 ± 0.16 cm^2^). The bacterial addition did not significantly affect the mycelial growth, both in the case of *T. borchii* and *T. melanosporum*, during all over the measurement period.

These results were also confirmed by the data of AGRs (Figure S3) during the exponential phase. The AGRs values of both these two species added with bacteria exhibited a non‐significant difference despite the control, according to *p* < 0.05 by Tukey's test. On the other hand, the AGR of the TMG5072 mycelium, about 1.66 ± 0.043 cm^2^/week, was significantly higher than other truffle species with or without bacteria addition, respectively: 0.69 ± 0.022 cm^2^/week for TBO5005_bac and 0.59 ± 0.024 cm^2^/week for TBO5005; 0.12 ± 0.011 cm^2^/week for TME2_bac and 0.22 ± 0.031 cm^2^/week for TME2.

### Bradyrhizobium *isolation*

The bacteria were unable to grow on mWPM without the mycelium, and no colony was formed after 1 month of incubation at both 22.5 and 28°C in the dark. Nevertheless, at 22.5°C, they can develop abundantly in a few days in the presence of the mycelium. Bacteria formed colonies within 10 days after inoculation in the absence of *T. magnatum* mycelium only on a selective medium (YMA) and with strictly specific conditions (28°C in the dark).

### Molecular characterisation and nucleotide sequence analyses

**FIGURE 3:**
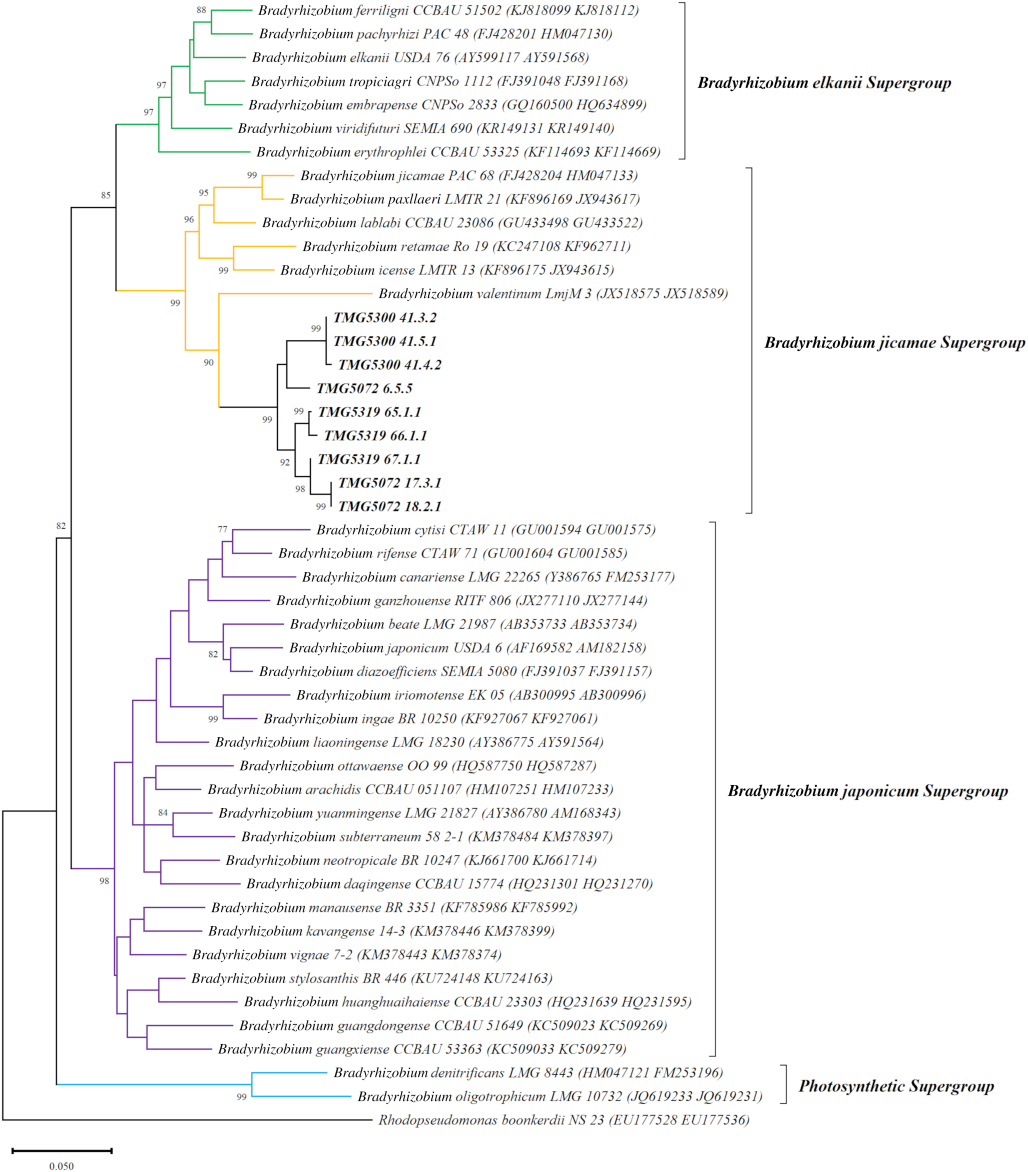
Maximum likelihood phylogeny based on concatenated *glnII*‐*recA* gene sequences showing the relationships between the nine *Tuber magnatum* bradyrhizobial strains isolated in this work and other members of the *Bradyrhizobium* genus. Accession numbers are indicated within brackets. Bootstrap values >75% are indicated at the nodes. Bar = 5 substitutions every 100 positions.

Preliminary analysis using *Bradyrhizobium*‐specific primers showed that all the bacterial colonies (10 from each *T. magnatum* strain) belonged to *Bradyrhizobium*. BLASTn analysis of the 16S rRNA gene sequences (Table S2) revealed that the bacterial isolates (three isolates from each *T. magnatum* strain) have the highest similarity (>99.8%) with *Bradyrhizobium* sp. strain SRL50 (MN134555), *Bradyrhizobium* sp. 170 (CP064703) and *Bradyrhizobium* sp. S12‐14‐2 (CP129212). ML analysis based on the 16S rRNA gene (Figure S4) placed the strains into a clade containing *Bradyrhizobium* spp. sequences from *T. borchii* (clone Cl‐19‐TB8‐II—AY599677) and *T. magnatum* (clone TM5_22—DQ303373; clone TM1_39—DQ303378) ascomata (Barbieri et al., 2005, 2007) but did not resolve the phylogenetic position of the bacteria isolated in this study within the *Bradyrhizobium* supergroups defined by Avontuur et al. (2019). In fact, the 16S rRNA gene sequences clustered together with species of both *Bradyrhizobium elkanii* supergroup (*Bradyrhizobium viridifuturi* and *Bradyrhizobium embrapense*) and *Bradyrhizobium jicamae* supergroup (*B. jicamae* and *Bradyrhizobium erythrophlei*). On the contrary, ML analysis of the concatenated *glnII* and *recA* gene dataset (Figure 3) grouped all bradyrhizobia strains isolated in this study in a monophyletic and well‐supported clade closely related to *Bradyrhizobium valentinum* (JX518575, JX518589) and species in the *B. jicamae* supergroup (Avontuur et al., 2019). The same topology of the ML tree was also obtained with NJ analysis (Figures S5 and S6).

**FIGURE 4:**
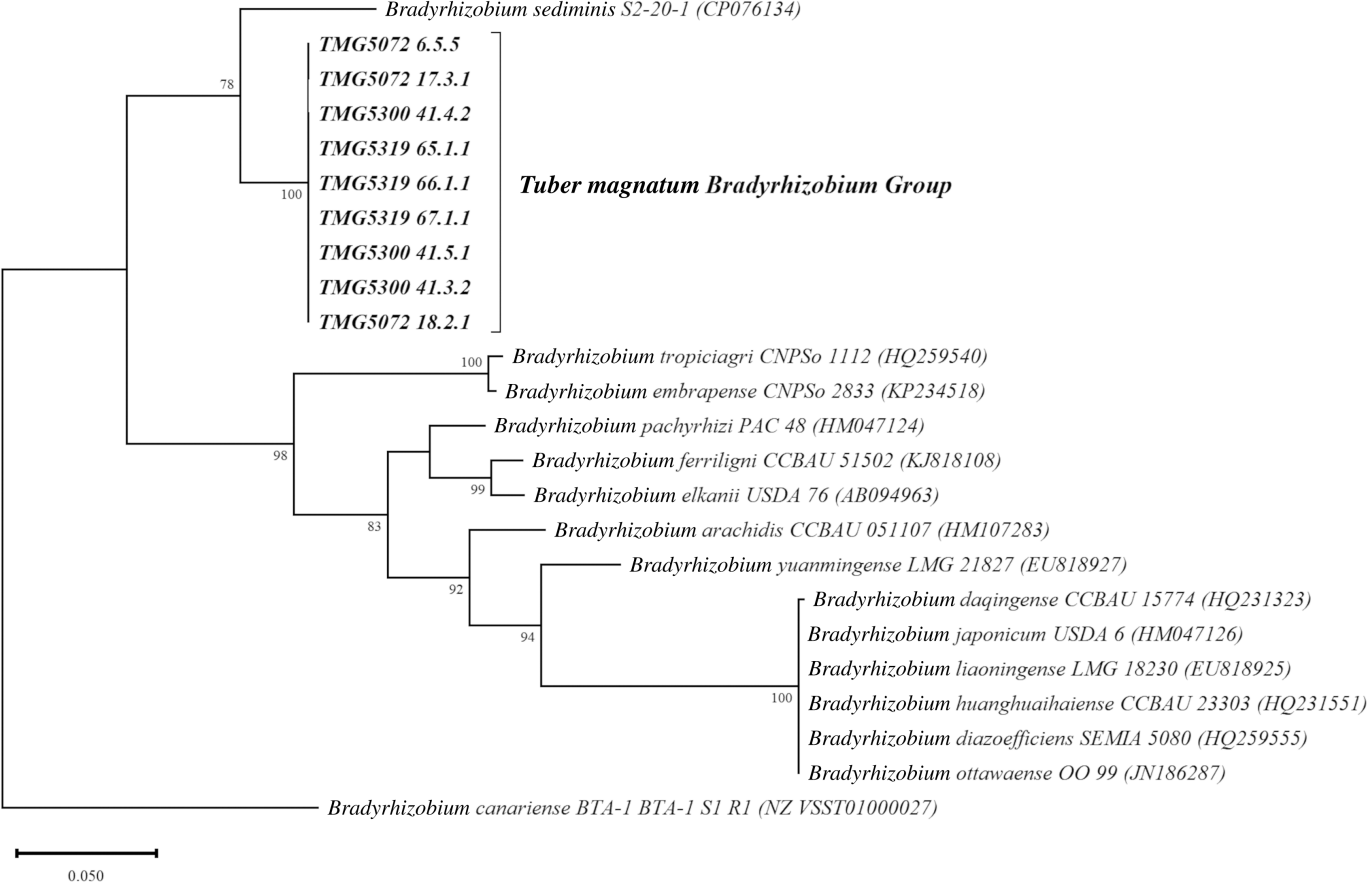
Maximum likelihood phylogeny of *nifH* gene sequences. Accession numbers are indicated within brackets. Bootstrap values >75% are indicated at the nodes. Bar = 5 substitutions every 100 positions.

The *nifH* phylogenetic tree (Figure 4) was congruent with the concatenated phylogeny inferred in this study by *glnII* and *recA* genes, placing all the nine *T. magnatum* bradyrhizobial strains in a separated clade, with a branch support of 100. Furthermore, these strains were strictly related to *Bradyrhizobium sediminis* S2‐20‐1 (CP076134).

The genetic diversity for *glnII* (Table S4) and *recA* (Table S5) genes within and between groups confirmed the belonging of *Bradyrhizobium* strains isolated in this group in the *B. jicamae* supergroup. In fact, uncorrected *p*‐distances between *T. magnatum* bradyrhizobia and the species of *B. jicamae* supergroup are on average always lower than the values calculated among the different supergroups for both *glnII* (0.08 versus >0.11) and *recA* (0.06 versus >0.08).

## DISCUSSION

Mycelia of different *Tuber* species have been successfully isolated by many authors and used for a variety of scientific purposes (Ceccaroli et al., 2001; Iotti et al., 2002, 2016; Leonardi et al., 2017; Li et al., 2012; Liu et al., 2009; Nadim et al., 2015; Poma et al., 1999; Saltarelli et al., 2003; Sbrana et al., 2002; Vahdatzadeh & Splivallo, 2018). However, viable and stable mycelial cultures of *T. magnatum* have been obtained for the first time only by this work, despite the numerous attempts made over the years. In this study, it was necessary to wait more than a month before the gleba fragments used as inoculum produced the first hyphae. This long lag phase may partly explain why *T. magnatum* mycelium has never been successfully isolated before. Furthermore, the recurrent development of bacteria on the inoculated gleba fragment might have led the researchers to discard isolation plates before the hyphal growth became evident (A. Zambonelli, personal communication, September 1, 2023). In our study, bacteria co‐isolated from the gleba proved to be essential for the growth of *T. magnatum* mycelium on mWPM, which is one of the most suitable media for *Tuber* mycelium (Iotti et al., 2002). As these bacteria were not able to improve the growth of *T. borchii* and *T. melanosporum* mycelia in the same conditions, it is possible to hypothesise a taxon‐specific dependence. Similarly, Le Roux et al. (2016) identified a specific interaction between bacterial strains belonging to the *Rhodopseudomonas* genus and the mycelia of *T. melanosporum* and *T. brumale*.

Even in the presence of the bacteria, all *T. magnatum* strains isolated in this study had slower growth than most of the saprotrophic cultivated basidiomycetes and ascomycetes (Badalyan et al., 2023; Puliga et al., 2022) but similar to other ectomycorrhizal fungi (Iotti et al., 2005). Considering the *Tuber* genus, the growth of the strain TMG5072 calculated as FCA was similar to that of *T. borchii* and higher than that of *T. melanosporum*. The mycelium of this strain appeared more branched (lowest HGU value) than the other strains although the HGU means were not statistically different. An increase in branching rate can be related to a more consistent growth, as demonstrated previously for *T. borchii* (Amicucci et al., 2010). Significant differences between strains were found for hyphal diameter and septal distance. In particular, the strain TMG5300 showed shorter and larger hyphal cells with respect to the other two strains but a lower branching rate. These morphological differences could be due to *T. magnatum* strain genetic differences or to the specific interaction with the bacteria lineages.

Molecular analyses showed that all the isolated bacteria belonged to *Bradyrhizobium*, which have previously been found to be common members of the bacterial community inhabiting the ascomata of *Tuber* spp. (Antony‐Babu et al., 2014; Barbieri et al., 2005, 2007, 2010, 2012; Benucci & Bonito, 2016; Citterio et al., 1995; Frey‐Klett et al., 2007; Graziosi et al., 2022; Marozzi et al., 2023; Monaco et al., 2021, 2022; Niimi et al., 2021a, 2021b; Pavić et al., 2013; Sillo et al., 2022). In particular, our 16S rRNA gene sequences clustered together with those of bradyrhizobia found in *T. borchii* and *T. magnatum* by Barbieri et al. (2005, 2007), with identities ranging from 98.2% to 99.7%. Similarly, the bacteria identified by Benucci and Bonito (2016) in the ascomata of several hypogeous ascomycetes (*Kalapuya brunnea* M.J. Trappe, Trappe & Bonito, *Leucangium carthusianum* (Tul. & C. Tul.) Paol., *Terfezia claveryi* Chatin, *Tuber indicum* Cooke & Massee, *T. melanosporum*, *Tuber lyonii* Butters, *Tuber gibbosum* Harkn. and *Tuber oregonense* Trappe, Bonito & P. Rawl.) and by Antony‐Babu et al. (2014) in *T. melanosporum* ascomata, have 16S rRNA gene sequence identities always >98.7%. Our findings confirm the hypothesis of the existence of a ubiquitous *Bradyrhizobium* taxon that is part of the core microbial community of *Tuber* ascomata (Benucci & Bonito, 2016). Considering the effects of these bacteria on *Tuber* mycelia, it can be assumed that the species of this taxon may promote gleba formation during ascoma maturation.

However, the 16S rRNA gene is too conserved in bradyrhizobia to discriminate between species and is not able to discriminate also between *Bradyrhizobium* and closely related genera (Willems et al., 2001). For this reason, we conducted a multilocus phylogeny using the genes *glnII* and *recA*, which allowed the best resolution of the evolutionary relationships of our nine bradyrhizobial strains within the *Bradyrhizobium* genus. The concatenated tree placed all nine strains in a strongly supported clade closely related to the species in the *B. jicamae* supergroup (Avontuur et al., 2019). Unfortunately, no sequences of *glnII* and *recA* from bradyrhizobia inhabiting truffle ascomata are available in GenBank. Intriguingly, the three strains from the ascoma TMG5300 were grouped together in the same subclade (bootstrap value = 99), whereas the six strains isolated from TMG5319 and TMG5072 were paraphyletic and divided into four independent lineages.

The analyses on genetic divergence within and among *Bradyrhizobium* supergroups seem to confirm the inclusion of *T. magnatum* bradyrhizobia group into the *B. jicamae* supergroup. The *B. jicamae* supergroup contains nitrogen‐fixing bacteria commonly associated with leguminous plants but also included soil free‐living bacteria (Avontuur et al., 2019; Ormeño‐Orrillo & Martínez‐Romero, 2019). We successfully amplified the *nifH* gene in all isolated strains and the tree generated with their sequences also grouped the nine *T. magnatum* bradyrhizobial strains in a separate clade with high bootstrap support. The *nifH* genes of *T. magnatum* bradyrhizobia appear to be evolutionarily closer to that of the free‐living, nitrogen‐fixing and non‐nodulating *B. sediminis* isolated from freshwater sediment (Jin et al., 2022) rather than the *nifH* sequences of the root symbiotic and nodulating species.

The detection of *nifH* genes in the ascoma‐inhabiting bacteria leads to speculation on their role in nitrogen nutrition of *T. magnatum* (Barbieri et al., 2010). Moreover, the inability of the co‐isolated bradyrhizobia to grow in pure culture on mWPM suggests a possible mutualistic interaction with *T. magnatum*. Le Roux et al. (2016) made the same assumption of symbiotic interaction between the mycelia of *T. melanosporum* and *T. brumale* and bacteria in the *Rhodopseudomonas*, although these authors did not detect *nifH* gene in the analysed bacterial strains.

The role of the mycelia of ectomycorrhizal fungi on bacterial growth was already demonstrated by Rigamonte et al. (2010) and it may be related to the production of trehalose and various polyols (e.g., mannitol and arabitol). Mannitol is the main carbon source of *Bradyrhizobium* within a selective medium (Keele Jr et al., 1969; Kuykendall, 2015). These sugars are commonly produced during the active growth of ectomycorrhizal basidiomycetes (Hampp & Schaeffer, 1999; Ineichen & Wiemken, 1992; Martin et al., 1984, 1998; Söderström et al., 1988) and ascomycetes (Martin et al., 1985, 1988) including *Tuber* spp. (Ceccaroli et al., 2003, 2011). For instance, the utilisation of mannitol and trehalose exudated by *Cantharellus cibarius* Fr. mycelium was common among *Pseudomonas* spp. from different environments (Rangel‐Castro et al., 2000; Rangel‐Castro, Danell, & Pfeffer, 2002; Rangel‐Castro, Danell, & Taylor, 2002). Also, the high level of trehalose accumulated in *Laccaria bicolor* (Maire) P.D. Orton hyphae chemoattracted and promoted the growth of the helper bacteria (Deveau et al., 2010).

The intriguing relationship between bacteria and mycorrhizal fungi might have arisen at the origin of their evolution and taxon‐specific interaction may have evolved over time (Frey‐Klett et al., 2007). Seneviratne and Jayasinghearachchi (2003) reported the mycelial colonisation of some soil fungi by *Bradyrhizobium* spp. The fungal partner provided a site for cell adhesion and its exudates served as a source of nutrition for bradyrhizobia. Microscopic observations carried out by Seneviratne and Jayasinghearachchi (2003) showed an extended hyphal adhesion between bacterial and fungal cells similar to that observed in *T. magnatum* mycelium, although in our case the interaction seems to be closer. In fact, *T. magnatum* and the co‐isolated bradyrhizobia benefit each other and cannot grow separately, at least in mWPM, suggesting a mutual dependency. This unique association seems to have become essential during the evolutionary process of this truffle species. The genetic closeness of the bacteria inhabiting truffle ascomata could be due to a co‐evolution process between some bradyrhizobia taxa and the *Tuber* spp. This co‐evolutional process between truffles and bradyrhizobia might have induced the differentiation of some bacterial strains extremely specific to certain *Tuber* spp.

The strict relations between *Bradyrhizobium* spp. and *T. magnatum* mycelial growth could have practical implications in truffle cultivation. In fact, the application of these bradyrhizobia could affect the success of the spore inoculation in the greenhouse mycorrhization process and, later, the maintenance of *T. magnatum* soil colonisation after plantation in the field. Moreover, cultivating in vitro *T. magnatum* mycelium opens up the possibility of obtaining mycorrhized plants with *T. magnatum* mycelial cultures.

For a better exploitation of the beneficial effects of these bacteria on *T. magnatum* cultivation, further studies will be necessary to characterise their physiology and the exact nature of their relationship with *T. magnatum* mycelium.

## AUTHOR CONTRIBUTIONS

**Simone Graziosi:** Conceptualization (equal); data curation (equal); investigation (lead); methodology (equal); software (lead); writing – original draft (equal); writing – review and editing (equal). **Federico Puliga:** Conceptualization (supporting); investigation (supporting); methodology (supporting); writing – review and editing (equal). **Mirco Iotti:** Data curation (equal); formal analysis (equal); methodology (equal); supervision (supporting); visualization (equal); writing – original draft (equal); writing – review and editing (equal). **Antonella Amicucci:** Conceptualization (supporting); methodology (equal); visualization (supporting); writing – review and editing (equal). **Alessandra Zambonelli:** Conceptualization (equal); funding acquisition (lead); project administration (lead); resources (lead); supervision (lead); validation (lead); visualization (equal); writing – original draft (equal); writing – review and editing (equal).

## CONFLICT OF INTEREST STATEMENT

The authors declare that they have no conflict of interest.

## Supporting information


**Data S1.** Supporting Information.

## Data Availability

All the sequences were deposited in GenBank database. All the other data are reported in the text and /or in supporting information.
