# Supplementary material for: In vitro interactions between Bradyrhizobium spp. and Tuber magnatum mycelium
Source: Environ Microbiol Rep. 2024 May 1;16(3):e13271. doi: 10.1111/1758-2229.13271 (PMC11062863; doi:10.1111/1758-2229.13271)
Supplement: Supplementary file 1 — Data S1. Supporting Information. [file EMI4-16-e13271-s001.docx]

**TITLE PAGE**

***In vitro* interactions between *Bradyrhizobium* spp. and *Tuber magnatum* mycelium**

**Running title: *Tuber magnatum* and bradyrhizobia interactions**

**Simone Graziosi^1*^, Federico Puliga^1^, Mirco Iotti^2^, Antonella Amicucci^3^, Alessandra Zambonelli^1^**

^1^Department of Agricultural and Food Sciences, University of Bologna, Viale G. Fanin 44, 40127, Bologna, Italy

^2^Department of Life, Health and Environmental Science, University of L'Aquila, Via Vetoio, 67100, Coppito, L'Aquila, Italy;

^3^ Department of biomolecular sciences, University of Urbino, v. Saffi, 2 – 61029 Urbino (PS), Italy

*Corresponding author: [simone.graziosi5@unibo.it](mailto:simone.graziosi5@unibo.it)

**Coauthor’ details:**

**Federico Puliga,** Department of Agricultural and Food Sciences, University of Bologna, Viale G. Fanin 44, 40127, Bologna, Italy, email: [federico.puliga2@unibo.it](file:///C:\Users\alessandr.zambonelli\AppData\Local\Microsoft\Windows\INetCache\Content.Outlook\KCL8SC3U\federico.puliga2@unibo.it)

**Mirco Iotti,** Department of Life, Health and Environmental Science, University of L'Aquila, Via Vetoio, 67100, Coppito, L'Aquila, Italy, email: [mirco.iotti@univaq.it](mailto:mirco.iotti@univaq.it)

**Antonella Amicucci,** Department of biomolecular sciences, University of Urbino, v. Saffi, 2 – 61029 Urbino (PS), Italy, email: [antonella.amicucci@uniurb.it](file:///C:\Users\alessandr.zambonelli\AppData\Local\Microsoft\Windows\INetCache\Content.Outlook\KCL8SC3U\antonella.amicucci@uniurb.it)

**Alessandra Zambonelli,** Department of Agricultural and Food Sciences, University of Bologna, Viale G. Fanin 44, 40127, Bologna, Italy, email: [alessandr.zambonelli@unibo.it](file:///C:\Users\alessandr.zambonelli\AppData\Local\Microsoft\Windows\INetCache\Content.Outlook\KCL8SC3U\alessandr.zambonelli@unibo.it)


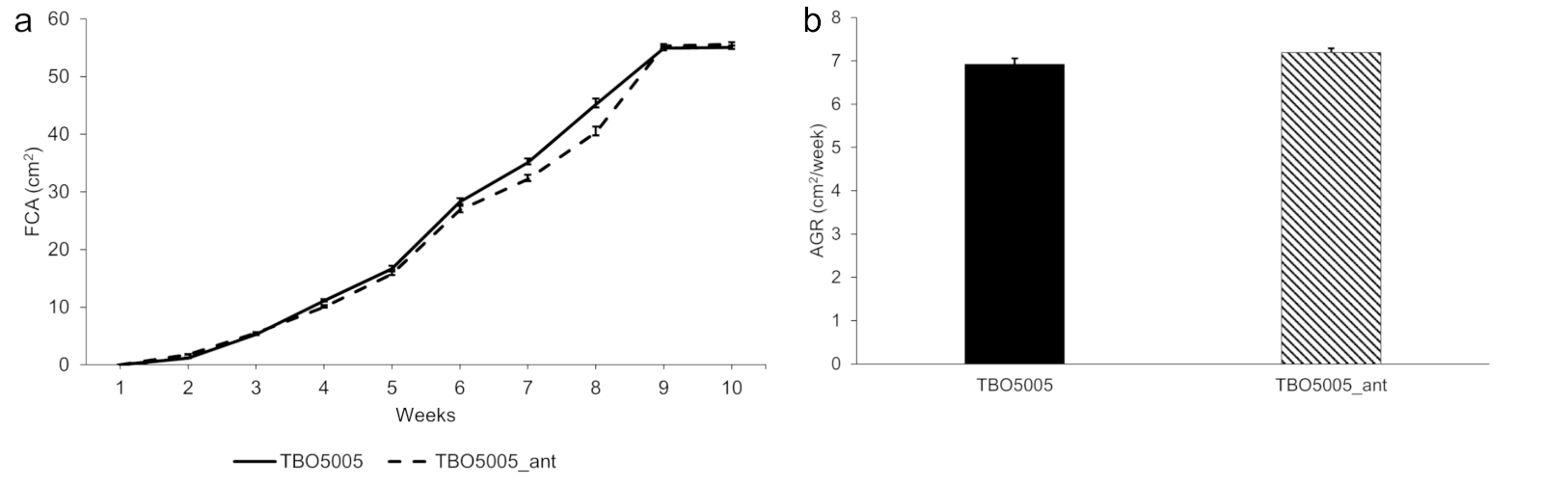


**Figure S1** Growth trend of the Fungal Colony Area (FCA) covered weekly by the mycelia of *Tuber borchii* 5005 comparing control and antibiotic addition (TBO5005, solid line; TBO5005_ant, dashed line) (a). Area Growth Rate (AGR) of *T. borchii* control (TBO5005) and *T. borchii* with antibiotic addition (TBO5005_ant) (b). No statistical difference was found between TBO5005 and TBO5005_ant AGRs.


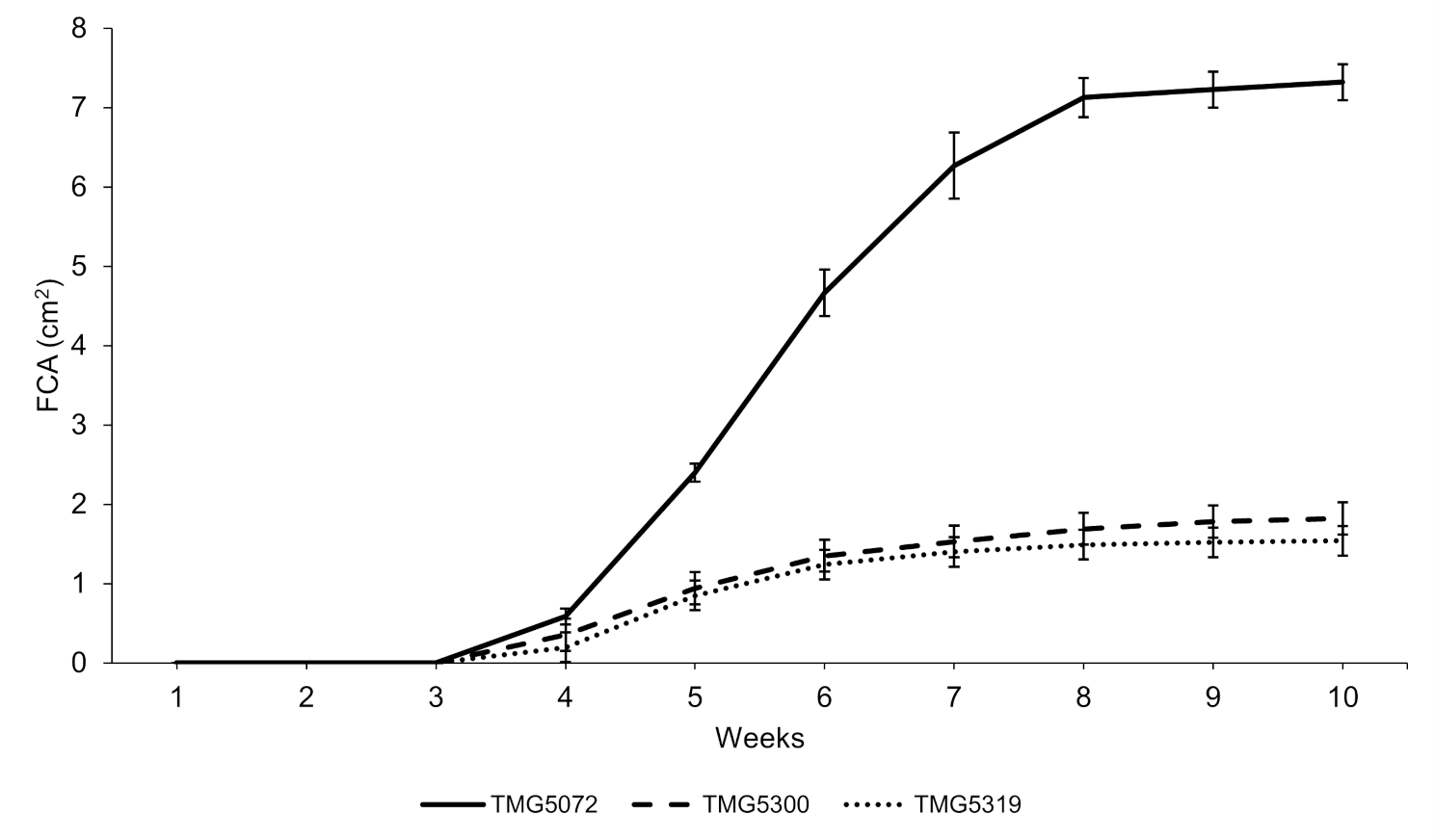


**Figure S2** Growth trend of the Fungal Colony Area (FCA) covered weekly by the mycelia of *Tuber magnatum* 5072, 5300, and 5319 strains (TMG5072 solid line; TMG5300, dashed line; TMG5319, dotted line).


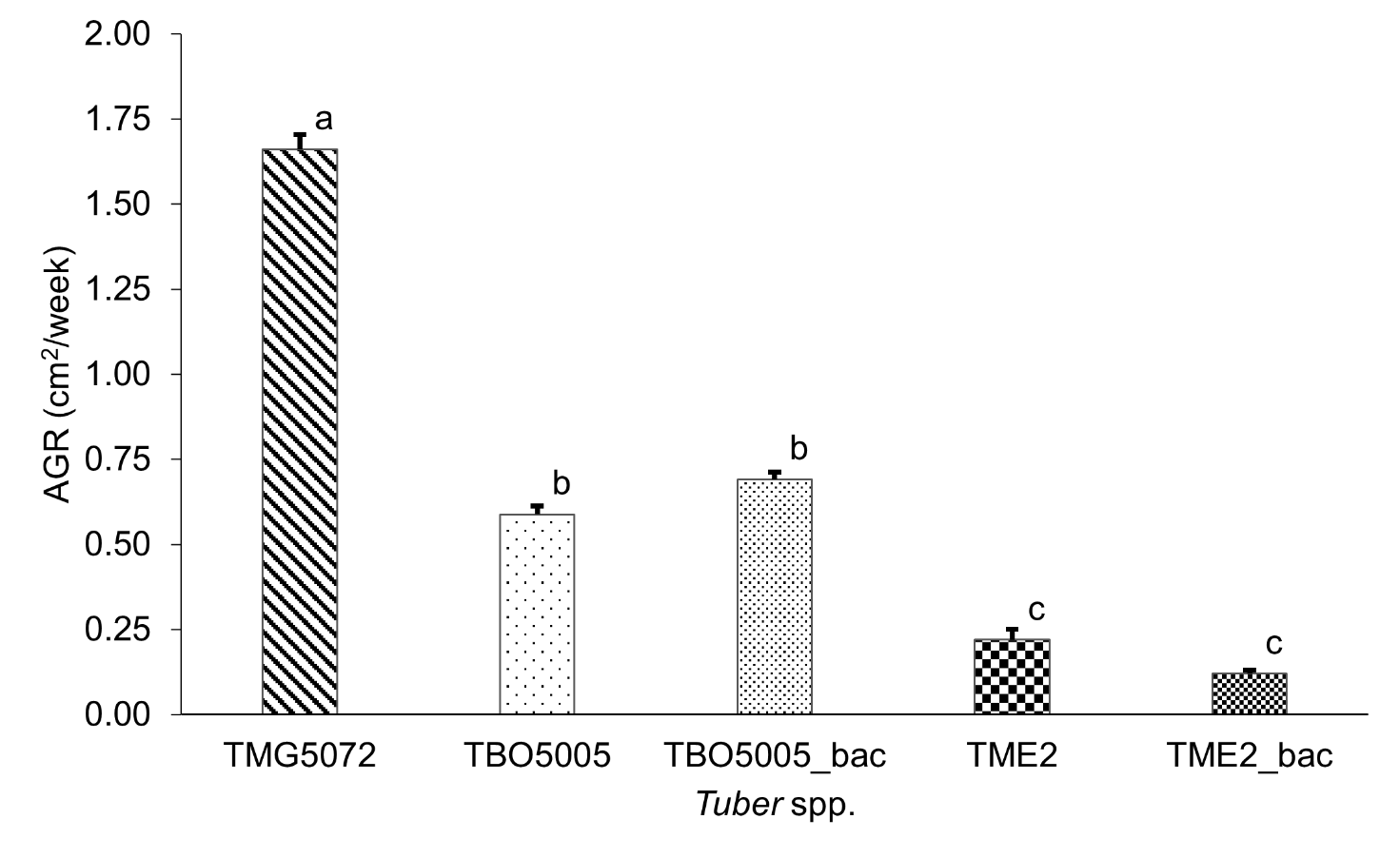


**Figure S3** Area Growth Rate (AGR) of *Tuber magnatum* (TMG5072), *Tuber borchii* control (TBO5005), *T. borchii* with bacterial addition (TBO5005_bac), *Tuber melanosporum* control (TME2), *T. melanosporum* with bacterial addition (TME2_bac). Letters represent different significant values according to Tukey's test with p < 0.05.


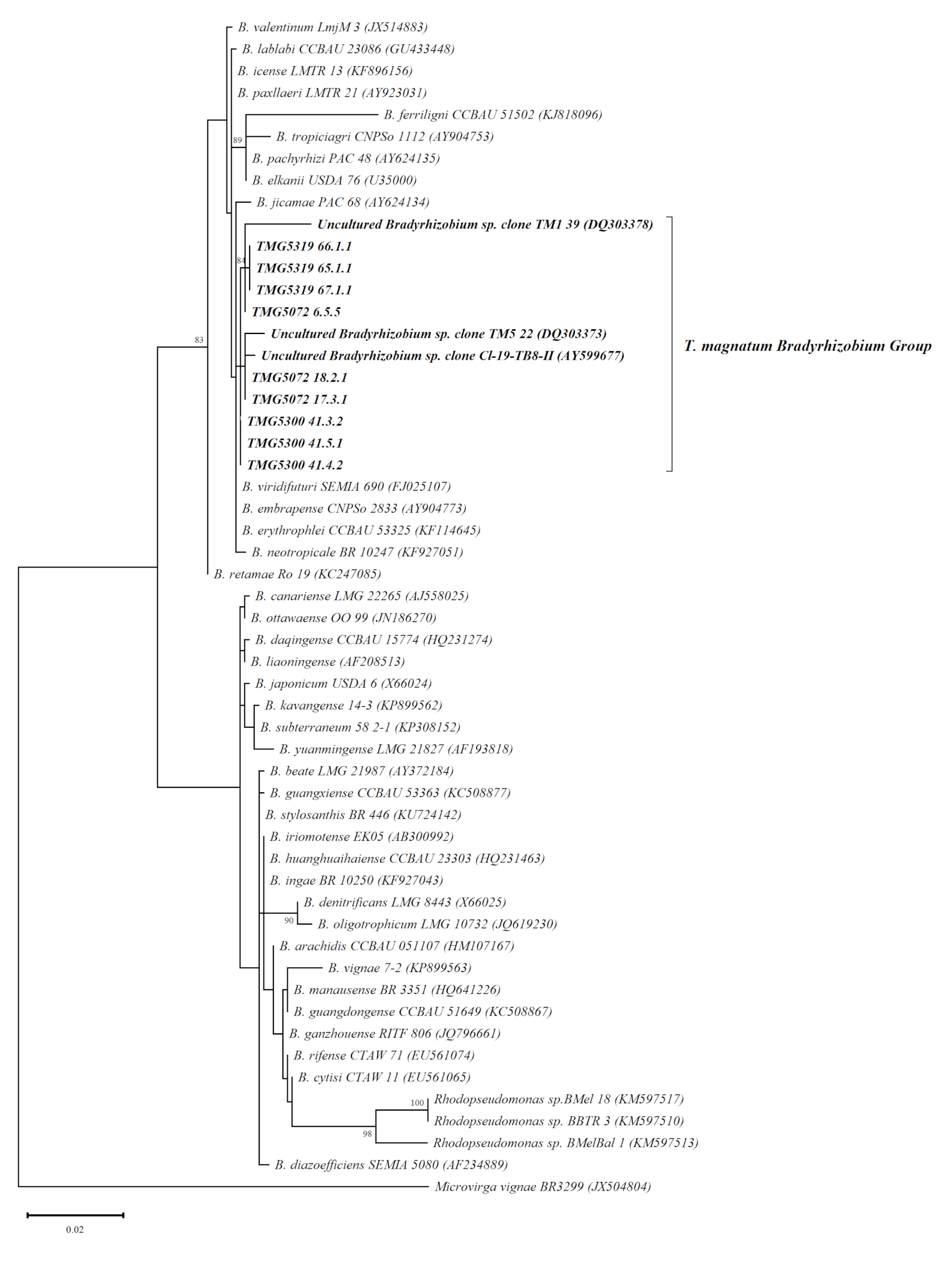


**Figure S4** Maximum likelihood phylogeny based on the 16Sr RNA gene sequences. Accession numbers are indicated within brackets. Bootstrap values >75% are indicated at the nodes. *Microvirga vignae* BR3299T was used as an outgroup. Bar = 2 substitutions every 100 positions.


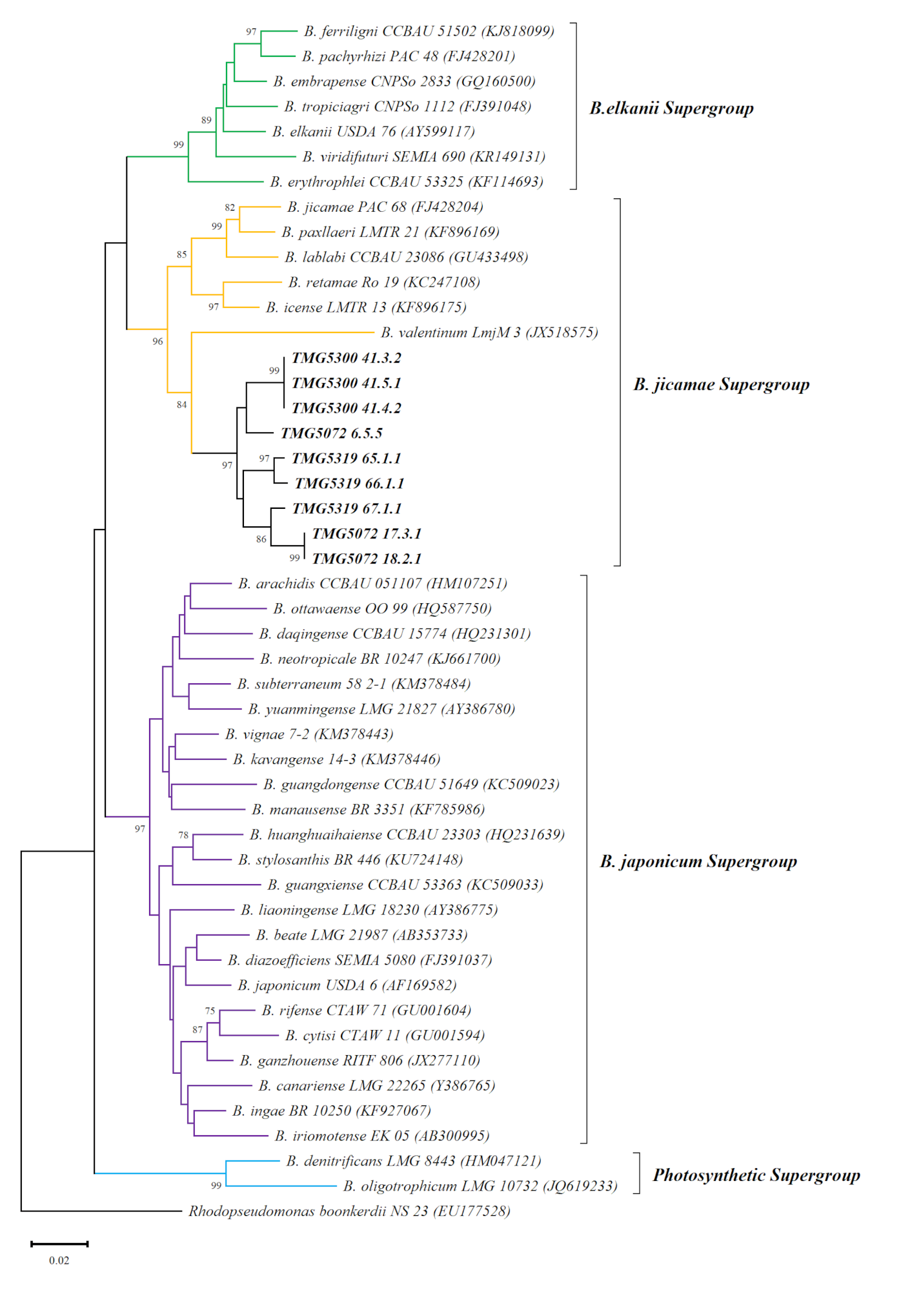


**Figure S5** Neighbor Joining phylogeny based on *glnII* gene sequences. Accession numbers are indicated within brackets. Bootstrap values >75% are indicated at the nodes. *Rhodopseudomonas boonkerdii NS 23* (EU177528) was used as an outgroup. Bar = 2 substitutions every 100 positions.


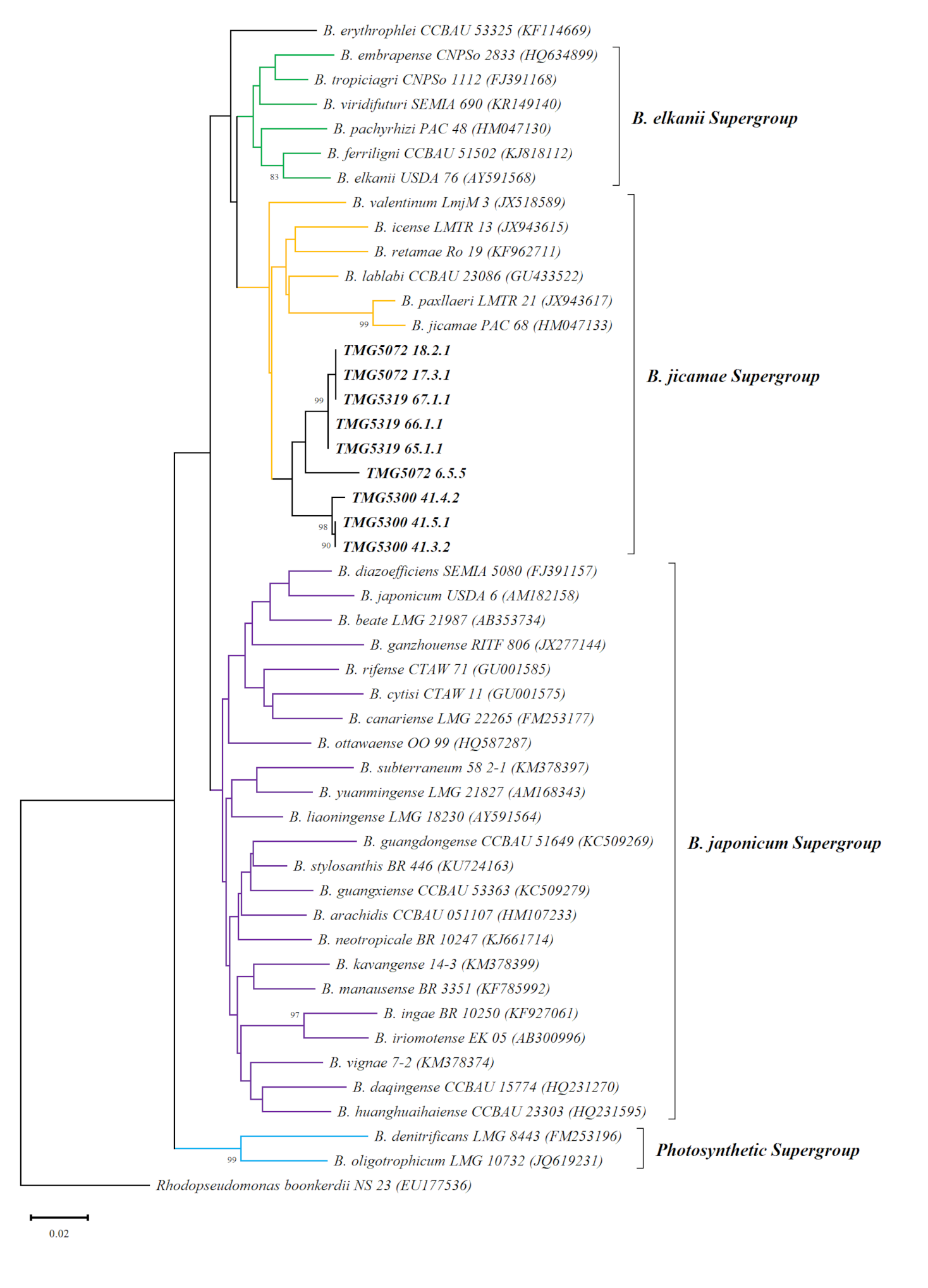
**Figure S6** Neighbor Joining phylogeny based on *recA* gene sequences. Accession numbers are indicated within brackets (*glnII* *recA*). Bootstrap values >75% are indicated at the nodes. *Rhodopseudomonas boonkerdii NS 23* (EU177536) was used as an outgroup. Bar = 2 substitutions every 100 positions

**Table S1** Oligonucleotide primers used and PCR cycling conditions to analyze bacterial isolates.

| Primer | Sequence (5’ – 3’) | Target gene (position) | PCR cycling conditions | Reference |
| --- | --- | --- | --- | --- |
| *27F* | AGAGTTTGATCCTGGCTCAG | 16S rRNA (8–27) | 95 °C 5 min; 30 x (95 °C 30 s, 52 °C 30 s, 72 °C 1 min), 72 °C 10 min | (Eden et al., 1991) |
| *1492R* | GGTTACCTTGTTACGACTT | 16S rRNA (1510–1492) |  |  |
| *BrdnaKf* | TTCGACATCGACGCSAACGG | *dnaK* (1411–1430) | 95 °C 2 min; 35 x (95 °C 45 s, 58 °C 30 s, 72 °C 1,5 min), 72 °C 7 min | (Menna et al., 2009) |
| *BrdnaKr* | GCCTGCTGCKTGTACATGGC | *dnaK* (1905–1885) |  |  |
| *nifseqF* | ATTCTGATCGTCGGTTGCG | *nifH* (100 – 118) | 95 °C 3 min; 35 x (95 °C 1 min, 59 °C 1 min, 72 °C 1 min), 72 °C 3 min | This study |
| *nifseqR* | GGATCTTCTCGGCAAGGC | *nifH* (722 – 742) |  |  |
| *recA63F* | ATCGAGCGGTCGTTCGGCAAGGG | *recA (63 – 85)* | 95°C 5 min; 32 x (94°C 45 s, 60°C 1 min, 74°C 1,5 min), 72°C 5 min | (Rivas et al., 2009) |
| *recA504R* | TTGCGCAGCGCCTGGCTCAT | *recA (504 – 523)* |  |  |
| *TSglnIIf* | AAGCTCGAGTACATCTGGCTCGACGG | *glnII* (13–38) | 95 °C 2 min; 35 x (95 °C 45 s, 58 °C 30 s, 72 °C 1,5 min), 72 °C 7 min | (Stępkowski et al., 2005) |
| *TSglnIIr* | SGAGCCGTTCCAGTCGGTGTCG | *glnII* (681–660) |  |  |

**Table S2** Nucleotide sequence characteristics of partial 16Sr RNA, *gln*II, *recA* and *nifH* obtained in this study, their origins, GenBank homologies and accession numbers.

| Bacterial isolate | Origin | Gene sequenced | Sequence length (bp) | Accession number | Closest BLASTn  (accession number) | Coverage (%) | Identity  (%) |
| --- | --- | --- | --- | --- | --- | --- | --- |
| TMG5072 6.5.5 | Glebal tissue TMG5072 | 16Sr RNA | 1288 | OR544968 | *Bradyrhizobium* sp. S12-14-2  (CP129212) | 100 | 99*.*92 |
|  |  | *glnII* | 623 | OR569722 | *Bradyrhizobium* sp. 170  (CP064703) | 100 | 97*.*91 |
|  |  | *recA* | 431 | OR569731 | *Bradyrhizobium* sp. strain SRL2  (MN159360) | 100 | 98.14 |
|  |  | *nifH* | 584 | OR569740 | *Bradyrhizobium* sp. S12-14-2  (CP129212) | 97 | 94*.*52 |
| TMG5072 17.3.1 | Glebal tissue TMG5072 | 16Sr RNA | 1326 | OR544969 | *Bradyrhizobium* sp. strain SRL50  (MN134555) | 100 | 100 |
|  |  | *glnII* | 623 | OR569723 | *Bradyrhizobium* sp. 170  (CP064703) | 100 | 96.31 |
|  |  | *recA* | 431 | OR569732 | *Bradyrhizobium* sp. gdr5 (KM247836) | 100 | 96.75 |
|  |  | *nifH* | 584 | OR569741 | *Bradyrhizobium* sp. S12-14-2  (CP129212) | 97 | 94*.*52 |
| TMG5072 18.2.1 | Glebal tissue TMG5072 | 16Sr RNA | 1288 | OR544971 | *Bradyrhizobium* sp. strain SRL50  (MN134555) | 100 | 100 |
|  |  | *glnII* | 623 | OR569724 | *Bradyrhizobium* sp. 170  (CP064703) | 100 | 96.15 |
|  |  | *recA* | 431 | OR569733 | *Bradyrhizobium* sp. gdr5 (KM247836) | 100 | 96.75 |
|  |  | *nifH* | 584 | OR569742 | *Bradyrhizobium* sp. S12-14-2  (CP129212) | 97 | 94*.*52 |
| TMG5300 41.3.2 | Glebal tissue TMG5300 | 16Sr RNA | 1288 | OR544972 | *Bradyrhizobium* sp. 170  (CP064703) | 100 | 100 |
|  |  | *glnII* | 623 | OR569725 | *Bradyrhizobium* sp. 170  (CP064703) | 100 | 98.07 |
|  |  | *recA* | 431 | OR569734 | *Bradyrhizobium* sp. gdr4 (KM247835) | 100 | 97.91 |
|  |  | *nifH* | 584 | OR569743 | *Bradyrhizobium* sp. S12-14-2  (CP129212) | 99 | 94*.*52 |
| TMG5300 41.4.2 | Glebal tissue TMG5300 | 16Sr RNA | 1320 | OR544973 | *Bradyrhizobium* sp. 170  (CP064703) | 100 | 100 |
|  |  | *glnII* | 623 | OR569726 | *Bradyrhizobium* sp. 170  (CP064703) | 100 | 98.07 |
|  |  | *recA* | 431 | OR569735 | *Bradyrhizobium* sp. gdr4 (KM247835) | 100 | 97.91 |
|  |  | *nifH* | 584 | OR569744 | *Bradyrhizobium* sp. S12-14-2  (CP129212) | 99 | 94*.*52 |
| TMG5300 41.5.1 | Glebal tissue TMG5300 | 16Sr RNA | 1320 | OR544970 | *Bradyrhizobium* sp. 170  (CP064703) | 100 | 100 |
|  |  | *glnII* | 623 | OR569727 | *Bradyrhizobium* sp. 170  (CP064703) | 100 | 98.07 |
|  |  | *recA* | 431 | OR569736 | *Bradyrhizobium* sp. gdr4 (KM247835) | 100 | 97.91 |
|  |  | *nifH* | 584 | OR569745 | *Bradyrhizobium* sp. S12-14-2  (CP129212) | 99 | 94*.*52 |
| TMG5319 65.1.1 | Glebal tissue TMG5319 | 16Sr RNA | 1098 | OR544965 | *Bradyrhizobium* sp. S12-14-2  (CP129212) | 100 | 99*.*82 |
|  |  | *glnII* | 623 | OR569728 | *Bradyrhizobium* sp. 170  (CP064703) | 100 | 97*.*75 |
|  |  | *recA* | 431 | OR569737 | *Bradyrhizobium* sp. gdr5 (KM247836) | 100 | 96.75 |
|  |  | *nifH* | 584 | OR569746 | *Bradyrhizobium* sp. S12-14-2  (CP129212) | 100 | 94*.*52 |
| TMG5319 66.1.1 | Glebal tissue TMG5319 | 16Sr RNA | 1098 | OR544966 | *Bradyrhizobium* sp. S12-14-2  (CP129212) | 100 | 99*.*82 |
|  |  | *glnII* | 623 | OR569729 | *Bradyrhizobium* sp. 170  (CP064703) | 100 | 97.43 |
|  |  | *recA* | 431 | OR569738 | *Bradyrhizobium* sp. gdr5 (KM247836) | 100 | 96.75 |
|  |  | *nifH* | 584 | OR569747 | *Bradyrhizobium* sp. S12-14-2  (CP129212) | 100 | 94*.*52 |
| TMG5319 67.1.1 | Glebal tissue TMG5319 | 16Sr RNA | 1098 | OR544967 | *Bradyrhizobium* sp. S12-14-2  (CP129212) | 100 | 99*.*82 |
|  |  | *glnII* | 623 | OR569730 | *Bradyrhizobium* sp. 170  (CP064703) | 100 | 97*.*75 |
|  |  | *recA* | 431 | OR569739 | *Bradyrhizobium* sp. gdr5 (KM247836) | 100 | 96.75 |
|  |  | *nifH* | 584 | OR569748 | *Bradyrhizobium* sp. S12-14-2  (CP129212) | 100 | 94*.*52 |

**Table S3** Strains used in this study for phylogenetic analysis.

| Strain names | Accession number  (*16SrRNA*; *glnII*; *recA*; *nifH*) | Original host species | Geographical origin | References |
| --- | --- | --- | --- | --- |
| *B. arachidis* CCBAU 051107 | (HM107167; HM107251; HM107233; HM107283) | *Arachis hypogaea* | China | (R. Wang et al., 2013) |
| *B. beate* LMG 21987 | (AY372184; AB353733; AB353734) | *Beta vulgaris* | Spain | (Islam et al., 2008; Rivas et al., 2004) |
| *B. canariense* LMG 22265 | (AJ558025; AY386765; FM253177; NZ_VSST01000027) | *Chamaecytisus proliferus* | Spain | (Jarabo-Lorenzo et al., 2003; Rivas et al., 2009; Vinuesa et al., 2005) |
| *B. cytisi* CTAW 11 | (EU561065; GU001594; GU001575) | *Cytisus triflorus* | Morocco | (Chahboune et al., 2011) |
| *B. daqingense* CCBAU 15774 | (HQ231274; HQ231301; HQ231270; HQ231323) | *Glycine max* | China | (J. Y. Wang et al., 2013) |
| *B. denitrificans* LMG 8443 | (X66025; HM047121; FM253196) | *Lablab purpureus* | China | (Han et al., 2008; Willems and Collins, 1992) |
| *B. diazoefficiens* SEMIA 5080 | (AF234889; FJ391037; FJ391157; HQ259555) | *Glycine max* | Brazil | (Chueire et al., 2003; Menna et al., 2009; Menna and Hungria, 2011) |
| *B. elkanii* USDA 76 | (U35000; AY599117; AY591568; AB094963) | *Glycine max* | USA | (Laguerre et al., 1997; Vinuesa et al., 2005) |
| *B. embrapense* CNPSo 2833 | (AY904773; GQ160500; HQ634899; KP234518) | *Desmodium heterocarpon* | Colombia | (Delamuta et al., 2015, 2012; Menna et al., 2006; Roma Neto et al., 2010) |
| *B. erythrophlei* CCBAU 53325 | (KF114645; KF114693; KF114669) | *Erythrophloeum fordii* | China | (Yao et al., 2015, 2014) |
| *B. ferriligni* CCBAU 51502 | (KJ818096; KJ818099; KJ818112; KJ818108) | *Erythrophleum fordii* | China | (Yao et al., 2015) |
| [*B. ganzhouense*](https://www.ncbi.nlm.nih.gov/pmc/articles/PMC4051118/) RITF 806 | (JQ796661; JX277110; JX277144) | *Acacia melanoxylon* | China | (Lu et al., 2014) |
| *B. guangdongense* CCBAU 51649 | (KC508867; KC509023; KC509269) | *Arachis hypogaea* | China | (Li et al., 2015) |
| *B. guangxiense* CCBAU 53363 | (KC508877; KC509033; KC509279) | *Arachis hypogaea* | China | (Li et al., 2015) |
| *B. huanghuaihaiense* CCBAU 23303 | (HQ231463; HQ231639; HQ231595; HQ231551) | *Glycine max* | China | (Zhang et al., 2011) |
| *B. icense* LMTR 13 | (KF896156; KF896175; JX943615) | *Phaseolus lunatus* | Perù | (Duran et al., 2014; López-López et al., 2013) |
| *B. ingae* BR 10250 | (KF927043; KF927067; KF927061) | *Inga laurina* | Brazil | (da Silva et al., 2014) |
| *B. iriomotense* EK 05 | (AB300992; AB300995; AB300996) | *Entada koshunensis* | Japan | (Islam et al., 2008) |
| *B. japonicum* USDA 6 | (X66024; AF169582; AM182158; HM047126) | *Glycine max* | USA | (Martens et al., 2007; Turner and Young, 2000; Willems and Collins, 1992) |
| *B. jicamae* PAC 68 | (AY624134; FJ428204; HM047133) | *Pachyrrhizus erosus* | Honduras | (Han et al., 2008; Ramírez-Bahena et al., 2009; Rodríguez-Navarro et al., 2004) |
| *B. kavangense* 14-3 | (KP899562; KM378446; KM378399) | *Vigna unguiculata* | Namibia | (Grönemeyer et al., 2014; Lasse Grönemeyer et al., 2015) |
| *B. lablabi* CCBAU 23086 | (GU433448; GU433498; GU433522) | *Lablab purpureus* | China | (Chang et al., 2011) |
| *B. liaoningense* LMG 18230 | (AF208513; AY386775; AY591564; EU818925) | *Glycine max* | China | (van Berkum and Fuhrmann, 2000; Vinuesa et al., 2005) |
| *B. manausense* BR 3351 | (HQ641226; KF785986; KF785992) | *Vigna unguiculata* | Brazil | (Silva et al., 2014) |
| *B. neotropicale* BR 10247 | (KF927051; KJ661700; KJ661714) | *Centrolobium paraense* | Brazil | (da Silva et al., 2014; Zilli et al., 2014) |
| *B. oligotrophicum* LMG 10732 | (JQ619230; JQ619233; JQ619231) | *Rice paddy soil* | Japan | Unpublished |
| *B. ottawaense* OO 99 | (JN186270; HQ587750; HQ587287; JN186287) | *Glycine max* | Canada | (Tang et al., 2012; Yu et al., 2014) |
| *B. pachyrhizi* PAC 48 | (AY624135; FJ428201; HM047130; HM047124) | *Pachyrhizus erosus* | Costa Rica | (Ramírez-Bahena et al., 2009; Rodríguez-Navarro et al., 2004) |
| *B. paxllaeri* LMTR 21 | (AY923031; KF896169; JX943617) | *Phaseolus lunatus* | Perù | (Duran et al., 2014; López-López et al., 2013; Ormeno-Orrillo et al., 2006) |
| *B. retamae* Ro 19 | (KC247085; KC247108; KF962711) | *Retama monosperma* | Morocco | (Guerrouj et al., 2013; Unpublished) |
| *B. rifense* CTAW 71 | (EU561074; GU001604; GU001585) | *Cytisus triflorus* | Morocco | (Chahboune et al., 2011) |
| *B. sediminis* S2-20-1 | CP076134 | Soil | China | Unpublished |
| *B. stylosanthis* BR 446 | (KU724142; KU724148; KU724163) | *Stylosanthes guianensis* | Brazil | (Delamuta et al., 2016) |
| *B. subterraneum* 58 2-1 | (KP308152; KM378484; KM378397) | *Arachis hypogaea* | Namibia | (Grönemeyer et al., 2015, 2014) |
| *B. tropiciagri* CNPSo 1112 | (AY904753; FJ391048; FJ391168; HQ259540) | *Neonotonia wightii* | Brazil | (Menna et al., 2009, 2006; Menna and Hungria, 2011) |
| *B. valentinum* LmjM 3 | (JX514883; JX518575; JX518589) | *Lupinus mariae-josephi* | Spain | (Duran et al., 2013) |
| *B. vignae* 7-2 | (KP899563; KM378443; KM378374) | *Vigna unguiculata* | Namibia | (Grönemeyer et al., 2016, 2014) |
| *B. viridifuturi* SEMIA 690 | (FJ025107; KR149131; KR149140) | *Centrosema pubescens* | Brazil | (Binde et al., 2009; Ferraz Helene et al., 2015) |
| *B. yuanmingense* LMG 21827 | (AF193818; AY386780; AM168343; EU818927) | *Lespedeza cuneata* | China | (Appunu et al., 2008; Stępkowski et al., 2007; Vinuesa et al., 2005; Yao et al., 2002) |
| *Microvirga vignae* BR 3299 | (JX504804) | *Vigna unguiculata* | Brazil | (Radl et al., 2014) |
| *Rhodopseudomonas boonkerdii* NS 23 | (na, EU177528; EU177536) | *Glycine max* soil | Thailand | (Noisangiam et al., 2010) |
| *Rhodopseudomonas* sp. BBTR 3 | (KM597510) | *Tuber brumale* | France | (Le Roux et al., 2016) |
| *Rhodopseudomonas sp.* BMelBal 1 | (KM597513) | *Tuber melanosporum* | France | (Le Roux et al., 2016) |
| *Rhodopseudomonas sp.*BMel 18 | (KM597517) | *Tuber melanosporum* | France | (Le Roux et al., 2016) |
| Uncultured *Bradyrhizobium* sp. clone Cl-19-TB8-II | (AY599677) | *Tuber borchii* | Italy | (Barbieri et al., 2005) |
| Uncultured *Bradyrhizobium* sp. clone TM1_39 | (DQ303378) | *Tuber magnatum* | Italy | (Barbieri et al., 2007) |
| Uncultured *Bradyrhizobium* sp. clone TM5_22 | (DQ303373) | *Tuber magnatum* | Italy | (Barbieri et al., 2007) |

Note: na = not available.

**Table S4** Genetic diversity among the *Bradyrhizobium* supergroups at the *glnII* region

|  | *B. elkani* S. | *B. japonicum* S. | *B. jicamae* S. | Photosynthetic S. | *T. magnatum* G. |
| --- | --- | --- | --- | --- | --- |
| *B. elkani* S. | 0.0478 |  |  |  |  |
| *B. japonicum* S. | 0.114 | 0.0587 |  |  |  |
| *B. jicamae* S. | 0.105 | 0.118 | 0.0689 |  |  |
| *Photosynthetic* S. | 0.135 | 0.131 | 0.134 | 0.0586 |  |
| *T. magnatum* G. | 0.112 | 0.115 | 0.0840 | 0.147 | 0.0298 |

Note: S. = supergroup; G. = group.

**Table S5** Genetic diversity among the *Bradyrhizobium* supergroups at the *recA* region

|  | *B. elkani* S. | *B. japonicum* S. | *B. jicamae* S. | Photosynthetic S. | *T. magnatum* G. |
| --- | --- | --- | --- | --- | --- |
| *B. elkani* S. | 0.0504 |  |  |  |  |
| *B. japonicum* S. | 0.0806 | 0.0722 |  |  |  |
| *B. jicamae* S. | 0.0773 | 0.0969 | 0.0578 |  |  |
| *Photosynthetic* S. | 0.109 | 0.113 | 0.122 | 0.0747 |  |
| *T. magnatum* G. | 0.0629 | 0.0858 | 0.0606 | 0.109 | 0.0222 |

Note: S. = supergroup; G. = group.
